# Electrochemical Characteristics of Glycerolized PEO-Based Polymer Electrolytes

**DOI:** 10.3390/membranes10060116

**Published:** 2020-06-05

**Authors:** Muhammad S. Mustafa, Hewa O. Ghareeb, Shujahadeen B. Aziz, M. A. Brza, Shakhawan Al-Zangana, Jihad M. Hadi, M. F. Z. Kadir

**Affiliations:** 1 Department of Chemistry, College of Science, University of Sulaimani, Qlyasan Street, Sulaimani 46001, Iraq; muhammad.mustafa@univsul.edu.iq (M.S.M.); hewa.ghareeb@univsul.edu.iq (H.O.G.); 2 Hameed Majid Advanced Polymeric Materials Research Lab, Physics, College of Science, University of Sulaimani, Qlyasan Street, Sulaimani 46001, Iraq; mohamad.brza@gmail.com; 3 Department of Civil Engineering, College of Engineering, Komar University of Science and Technology, Sulaimani 46001, Iraq; 4 Manufacturing and Materials Engineering Department, Faculty of Engineering, International Islamic University of Malaysia, Kuala Lumpur 50603, Malaysia; 5 Department of Physics, College of Education, University of Garmian, Kalar 46021, Iraq; shakhawan.al-zangana@garmian.edu.krd; 6 Kurdistan Technical Institute, Sulaimani 46001, Iraq; jihad.chemist@gmail.com; 7 College of Engineering, Tishk International University, Sulaimani 46001, Iraq; 8 Centre for Foundation Studies in Science, University of Malaya, Kuala Lumpur 50603, Malaysia; mfzkadir@um.edu.my

**Keywords:** glycerolized PEO electrolyte, dielectric properties, electric modulus study, impedance, LSV and TNM, EDLC and CV

## Abstract

In this article, poly(ethylene oxide)-based polymer electrolyte films doped with ammonium iodide (NH_4_I) and plasticized with glycerol were provided by a solution casting method. In the unplasticized system, the maximum ionic conductivity of $3.96\times 10^{-5}\;$S cm^−1^ was achieved by the electrolyte comprised of 70 wt. % PEO:30 wt. % NH_4_I. The conductivity was further enhanced up to $\;(1.77\times 10^{-4}$ S cm^−1^) for the plasticized system when 10 wt. % glycerol was added to the highest conducting unplasticized one at ambient temperature. The films were characterized by various techniques to evaluate their electrochemical performance. The results of impedance spectroscopy revealed that bulk resistance (R_b_) considerably decreased for the highest plasticized polymer electrolyte. The dielectric properties and electric modulus parameters were studied in detail. The LSV analysis verified that the plasticized system can be used in energy storage devices with electrochemical stability up to 1.09 V and the TNM data elucidated that the ions were the main charge carrier. The values of the ion transference number (t_ion_) and electron transfer number (t_el_) were calculated. The nonappearance of any redox peaks in the cyclic voltammograms indicated that the chemical reaction had not occurred at the electrode/electrolyte interface.

## 1. Introduction

So as to improve energy storage systems with efficient energy conservation and a low greenhouse gas emission, many attempts have been made of late to achieve high-performance components for next generation batteries [1,2,3,4,5,6,7,8,9]. Lately, electric double-layer capacitors (EDLCs)have attracted more attention due to having unique characteristics, such as higher energy density, durability, reversibility, quick charge–discharge rate, and improved safety, which makes them striking for a broad range of applications [10]. As a complementary technology device, EDLCs can be deliberated as a potential alternative to conventional lithium batteries [11,12,13,14]. In this type of electrochemical capacitor, the mechanism of energy storage is based on the accumulation of charge at the surface of a carbon electrode that converts into potential energy [15,16,17]. The electricity is physically stored by producing an electrical double-layer involving an adsorbed layer of anions and cations at the electrode/electrolyte interfaces [18]. The electrolyte is a crucial and substantial component in supercapacitors and has an important role in balancing as well as transporting charges between the electrodes. The interaction among the electrodes and electrolytes and in all electrochemical mechanisms considerably impacts the active materials’ internal structure as well as the electrolyte and electrodes’ interface states [19,20]. At the same time, several active materials have been utilized for making EDLC electrode, for instance graphite [21], aerogel [22], carbon nano-fiber [23] and activated carbon [24]. The activated carbon has unique characteristics, such as cost-effectiveness, relatively high electronic conductivity, and satisfactory chemical stability [25,26]. From the surface area point of view, activated carbon delivers a large double-layer for ion accumulation at the interfacial region as an energy storage mechanism. It is well-known that in this device, the energy storage process composes a non-Faradaic reaction [27,28].

Among the electrochemical device components, one crucial component is the electrolyte. Liquid electrolytes are replaced by polymer ones, since the former suffers from several major shortcomings of flammability, electrolyte leakage, as well as chemical instability [29]. In addition, the benefits of solid polymer electrolytes (SPEs), for instance, exceptional electrochemical stability, flexibility, high specific energy, ease of processing into thin films, and leak-proof nature make them promising for a variety of solid state electrochemical device applications containing supercapacitors, fuel cells, batteries, electrochromic devices, and chemical sensors [10,30]. Poly (ethylene oxide) (PEO) as a host polymer played a vital role in the preparation of SPEs by mixing with different alkali metal salts for some of the abovementioned device applications [31,32,33,34]. In such systems, the alkali metal ions interact with the ether oxygens in the chains of PEO, and, thus, their mobility is significantly influenced by the movement of the polymer segments. Polymer electrolytes usually contain both crystalline and amorphous phases. It has been recognized that most of the ion conduction occurs in the amorphous phase [35]. Nowadays, the main disadvantage of PEO-based electrolytes is their limited ionic conductivity (i.e., <10^−5^ S cm^−1^ at room temperature) and the reason for low conductivity below about 65 °C is polymer crystallinity [36]. Typically, a general method to address the ionic conductivity depends on the liquid plasticizer addition, such as ethylene carbonate or glycerol, which, by decreasing the crystalline structure inside the polymer, can be enhanced [37,38]. Accordingly, Pawlicka et al. [39] documented an enhancement of ionic conductivity from 10^−8^ to 10^−4^ S cm^−1^ when glycerol plasticizer was used in their electrolyte system. In the present work, PEO-based films consisting of glycerol-plasticized and unplasticized PEO:NH_4_I systems were characterized by electrochemical impedance spectroscopy, dielectric analysis, electrical modulus analysis, transference number measurements, linear sweep voltammetry, and cyclic voltammetry. This is to study the ionic conductivity, electrical energy behavior, the main charge carrier, and electrochemical stability, and performance of the present systems.

## 2. Experimental Methodology

### 2.1. Materials

All chemical materials and solvents were directly used without any purification. They included poly (ethylene oxide) powder (PEO, molecular weight ˃ 500,000 g mol^−1^, Alfa Aesar, lancashire, United Kingdom), glycerol (C_3_H_8_O_3_, 99.5% purity, Merck, Darmstadt, Germany), ammonium iodide (NH_4_I, 99% purity, Merck) and acetonitrile (ACN, 99.9% purity, Alpha Chemika, Mumbai, India).

### 2.2. Sample Preparation

A typical solution casting procedure utilized for the preparation of a series of unplasticized and plasticized PEO systems were as follows. For the unplasticized PEO system, three individual solutions have prepared by dissolving 1g of PEO in 40 mL of acetonitrile, and three other solutions were prepared separately by dissolving different weight ratios ofNH_4_I(i.e., 10, 20 and 30 wt. %) in 10 mL of the same solvent. The latter solutions were then added to the polymer solutions under continuous stirring at ambient temperature until clear homogenous solutions were achieved. Eventually, each solution was poured into several clean and dry Petri dishes (8 cm in diameter) and covered with filter paper to allow evaporating the solvent completely at room temperature. The resulting unplasticized PEO systems were coded as PEOH1, PEOH2, and PEOH3incorporated with 10, 20, and 30 wt. % NH_4_I, respectively. The same procedure abovementioned was repeated for the preparation of a plasticized PEO system by adding 10 wt. % of glycerol plasticizer to a solution containing 1g PEO and 30 wt. % of NH_4_I, coded as PEOH4. Table 1 shows the composition of the prepared PEO films.

### 2.3. Electrochemical Impedance Spectroscopy (EIS)

Impedance measurements for all PEO films were implemented using the LCR meter (HIOKI 3531Z HITESTTER, Tokyo, Japan) controlled by computer in the frequencies from 50 Hz to 5 MHz. Each film was sandwiched between two stainless steel blocking electrodes to analyze real and imaginary parts of impedance spectra at surrounding temperature. The stainless steel electrodes are inert electrodes and used for blocking ions of the polymer electrolytes.

### 2.4. LSV and TNM Measurements

The decomposition voltage, i.e., electrochemical stability, of the largest conducting plasticized system (i.e, PEOH4) was recorded by an LSV (Digi-IVY DY2300 potentiostat, Neware, Shenzhen, China) at surrounding temperature. The potential range was in between 0 and 2.5 V and the sweep rate was 10 mV s^−1^. The DC polarization technique was applied to analyze the transference number of the PEOH4 at room temperature and applied voltage of 0.2 V. Similarly, the PEO film was introduced among two stainless steel electrodes in a Teflon holder. The current was scanned against time using the V&A instrument (Neware, Shenzhen, China, DP3003 digital DC power).

### 2.5. EDLC Fabrication

Initially, the dry mix procedure was carried out for preparing the EDLC electrode. Planetary ball miller was used for dry mixing carbon black (CB) (0.25 g) and activated carbon (AC) (3.25 g). Later on, they were mixed with the N-methyl pyrrolidone (NMP) (15 mL) and polyvinylidene fluoride (PVdF) (0.5 g) solution. After stirring the mixture for a few hours, a thick black solution appeared. The doctor blade was applied for coating this black solution on a current collector (i.e., aluminum foil). The resulting coated aluminum foils were dried at 60 °C in the oven and further left in a desiccator in order to keep them with 2.01 cm^2^ area and thickness of ~0.02 cm. The cell arrangement in the EDLC is as follows:

AC electrode | maximum conducting SPE | AC electrode.

A CR2032 coin cell was filled with this cell. First of all, a Digi-IVY DY2300 potentiostat (10 mV/s scan rate) was used for conducting the cyclic voltammetry (CV) evaluation on the created EDLC.

### 2.6. CV Measurements

The CV study for the PEOH4 was carried out in the potential range of 0 to 0.9 V at different sweep rates of 10, 20, 50, and 100 mV s^−1^. The current was recorded as a function of potential using a Digi-IVY DY2300 potentiostat (Neware, Shenzhen, China). During the CV measurement, when the potential difference is zero, the current exists in the system that might be resulted from hysteresis. Furthermore, the complete cycle is taken for CV measurement and some current is maintained. However, the current is close to zero at very low scan rates, for example, below 5 mV s^−1^.

## 3. Results and Discussion

### 3.1. Electrical Properties

#### 3.1.1. Dielectric and Electric Modulus Study

**Figure 1:**
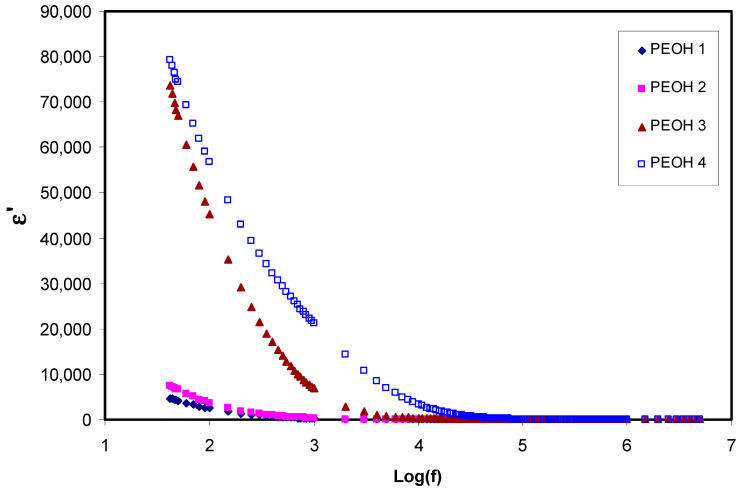
Real part of dielectric constant ($\varepsilon^{\prime }$) against frequency for un-plasticized PEOH1, PEOH2, PEOH3 and plasticized PEOH4 at room temperature.

**Figure 2:**
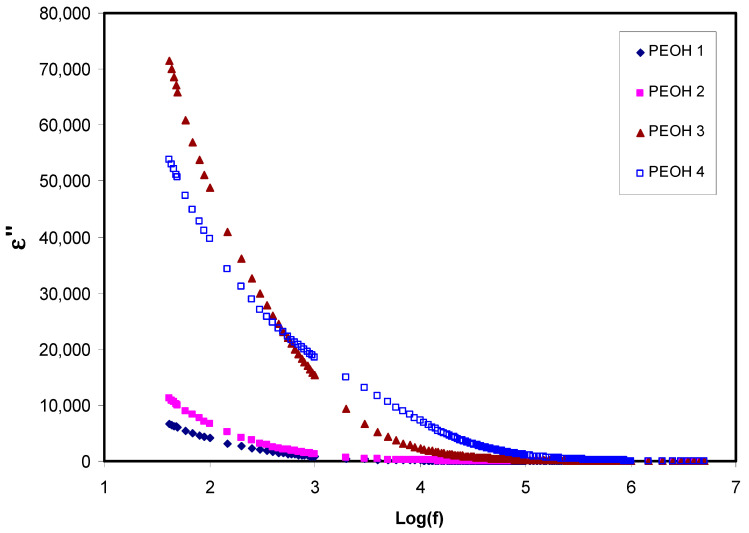
Imaginary part of dielectric loss ($\varepsilon^{\prime \prime }$) against frequency for unplasticized PEOH1, PEOH2, PEOH3 and plasticized PEOH4 at room temperature.

Ions conducting solid electrolytes are found to be the heart of electrochemical devices, and thus, the study of electrical properties, such as DC conductivity, dielectric properties, and electric modulus is crucial to understand the ion transport mechanism [40,41,42]. The efficiency of the polymer electrolyte films, with respect to the storage of the electrical energy and the loss of electrical energy as the heat, was represented by dielectric constant and dielectric loss, respectively, as given by the following equations [43,44,45]:

(1) $$\varepsilon^{\prime }=\frac{Z^{\prime \prime }}{\omega \;C_{o}({Z^{\prime }}^{2}+{Z^{\prime \prime }}^{2})}$$

(2) $$\varepsilon^{\prime \prime }=\frac{Z^{\prime }}{\omega \;C_{o}({Z^{\prime }}^{2}+{Z^{\prime \prime }}^{2})}$$

where $\varepsilon^{\prime }$, $\varepsilon^{\prime \prime }$ are dielectric permittivity($\;\varepsilon^{*}$) real and imaginary parts, which are also called dielectric constant and dielectric loss, respectively. $Z^{\prime }\;\mathrm{and}\;Z^{\prime \prime }$ are real and imaginary components of impedance, respectively. ω  is the circular frequency, and $C_{o}$ is the vacuum capacitance [45]. Figure 1 and Figure 2, show the$\;\varepsilon^{\prime }$ and $\varepsilon^{\prime \prime }$ against frequency, respectively, for all the samples at ambient temperature. As can be seen, the variations of both$\;\varepsilon^{\prime }$ and $\varepsilon^{\prime \prime }$ with respect to frequency could be ascribed to the space region creation at the interfaces of electrolyte and electrode, which is called non-Debye like behavior. In this regard, the present space charge regions were clarified in terms of ion diffusion. It can be noted that in the low dispersion frequency region, both$\;\varepsilon^{\prime }$ and $\varepsilon^{\prime \prime }$ displayed high values for all samples because of the high charge collection at the interfaces of electrodes-electrolytes [46]. Thus, the magnitude of these values increases upon increasing salt content (NH_4_I), demonstrating increment of the amount of charge storage, and density of mobile ions in the samples [47]. Furthermore, the conductivity and dielectric constant were improved with the addition of glycerol plasticizer to the PEO:NH_4_I sample (PEOH4). This leads to the creation of more free mobile ions by enhancing salt dissociation, and thus, the conductivity was improved since no relaxation peaks appeared on both graphs [48]. It can also be observed that from Figure 1 and Figure 2, the values of $\varepsilon^{\prime }$ and $\varepsilon^{\prime \prime }$ decrease with increasing frequency until reaching a region to become almost constant with frequency. The constant values at the high frequency region are attributed to the fast period of reversal of the electrical field, and the resulting charge carrier did not have enough time to orient themselves in the direction of the applied electric field. Consequently, no surplus ion diffusion in the direction of the field occurs [49,50]. Clearly, peaks are not observed in the spectra of dielectric loss, and this indicates that dipolar motion was masked by ion movement [51,52].

It is well established that ion conductivity is a hard subject in polymer electrolytes. The electric modulus is the electric permittivity reciprocal and is employed to examine the behavior of a dielectric property of polymer caused by the relaxation of ions. In polymer electrolytes, charge buildup suppression closing the electrodes is interrelated to the influence of electrode polarization and can be minimized through the electric modulus study [53,54,55]. Further study of dielectric behavior of the polymer electrolyte systems can be accomplished through dielectric modulus analysis as represented by the following equations [55,56]:

(3) $$M^{\prime }=\omega C_{o}Z_{i}$$

(4) $$M^{\prime \prime }=\omega C_{o}Z_{r}$$

**Figure 3:**
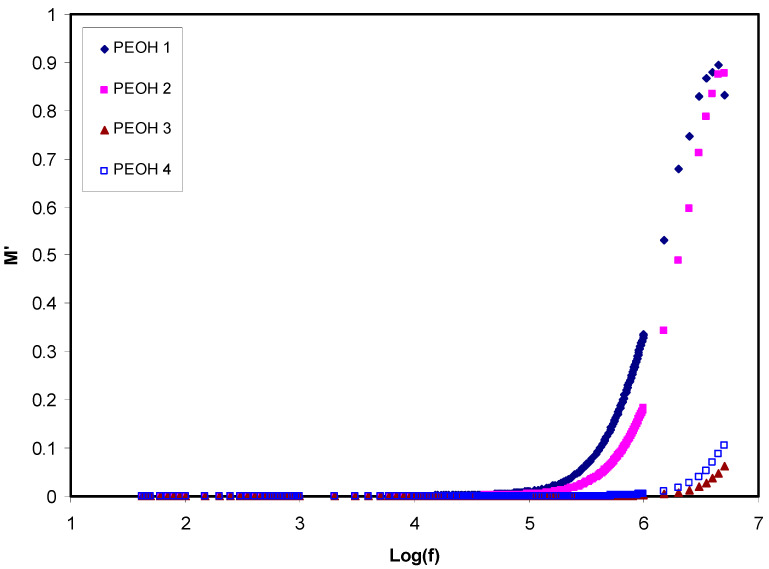
Part of dielectric constant loss ($M^{\prime }$) against frequency for un-plasticized PEOH1, PEOH2, PEOH3 and plasticized PEOH4 at room temperature.

**Figure 4:**
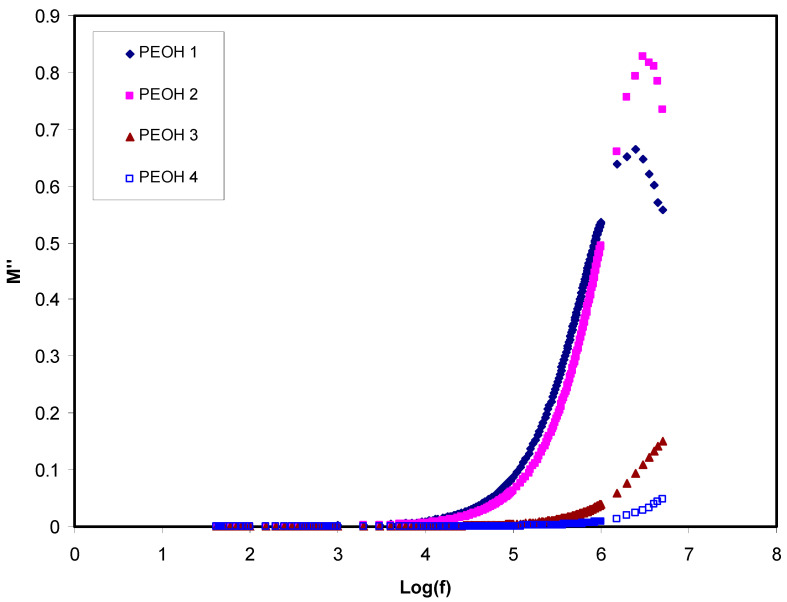
Imaginary part of dielectric constant loss ($M^{\prime \prime }$) against frequency for un-plasticized PEOH1, PEOH2, PEOH3 and plasticized PEOH4 at room temperature.

Hence, $M^{\prime }$ is the real part and $M^{\prime \prime }$ is the imaginary part of the electrical modulus [42]. Figure 3 and Figure 4 illustrate$\;M^{\prime }$ and $M^{\prime \prime }$ as a function of frequency for all samples at ambient temperature, respectively. The values of $M^{\prime }$ and $M^{\prime \prime }$ were augmented in the high frequencies area, while making a long tail at the low frequencies area. The latter one can be explained by the polarization phenomenon which gives a large capacitance associated with electrodes and a high dielectric constant. Some relaxation peaks observed due to bulk effects at high frequencies in the modulus formalism demonstrates that the films of polymer electrolyte are mainly ionic conductors [55,57]. As can be seen from the figures, part of the relaxation peaks at high frequency regions disappeared when the plasticizer was used in the high conducting polymer electrolyte film (PEOH4). This can also be interpreted by increasing conductivity via a high mobile ion concentration. On the other hand, the incorporation of glycerol caused a rise in the mobility of charge transport ions by decreasing the local viscosity around the ions.

#### 3.1.2. Impedance Spectroscopy Study

**Figure 5:**
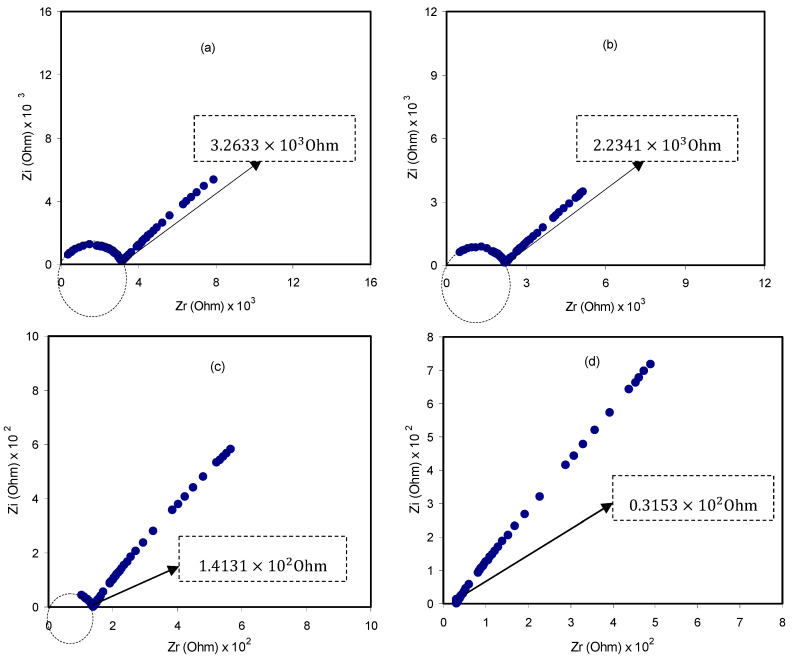
The room temperature impedance plots for (**a**) PEOH1; (**b**) PEOH2; (**c**) PEOH3 and (**d**) PEOH4.

The polymer electrolyte ionic conductivity was studied by the impedance spectroscopic technique. The ionic conductivity mainly relies on the concentration charge carrier and their mobility, represented by the following equation [58,59]:

(5) $$\sigma =\sum_{i}n_{i}q_{i}\mu_{i}$$

where $n_{i}$ is the mobile ions concentration, $q_{i}$ is the charge of a mobile carrier, and $\mu_{i}$ is the charge carrier mobility. Besides, the DC ionic conductivity ($\sigma_{dc})$ for all polymer electrolyte films was calculated by:

(6) $$\sigma_{dc}=\frac{l}{R_{b}A}$$

where l  stands for the film thickness, $R_{b}$ stands for the bulk resistance, and A  stands for the known electrolyte film area. Figure 5 depicts impedance spectra (spectra between impedance real and imaginary parts) for all samples at room temperature. Such impedance plots consist of two obvious regions, a high frequencies semicircle and a low frequencies spike (straight line) region. The spike region is related to the free charge buildup at the interfaces between solid electrolyte and electrode surface, resulting in the electric double-layer capacitor creation [60]. The semicircle at a higher region of frequency is due to the bulk conductivity of the polymer electrolytes [61]. From the Figure 5a–c, the tendency of a spike at low frequency range and a decrease in the diameter of the semicircle at the high frequency range attributed to the existence stainless steel blocking electrodes, i.e., blocking double-layer capacitance at the blocking electrodes [62]. Moreover, as the concentration of the salt increases especially at 30 wt. %, the semicircle gradually shrinks at the high frequency region; as a result, the polymer electrolyte films bulk resistance$\;(R_{b})$ declines, and consequently, ionic conductivity increases [63]. The semicircular portion disappearance in Figure 5d indicates that the entire conductivity is primarily as a result of the migration of ions at the largest salt amount, which is facilitated by the existence of glycerol as a plasticizer. The value of $R_{b}$ can be found accurately from the intercept of the straight line on the real axis of the impedance curve. Table 2 shows the calculated DC ionic conductivity for all polymer electrolyte films at room temperature. It can be observed that the ionic conductivity improved with increment salt concentration, which pertains to the escalation in the charge carrier amount. Significant enhancement in ionic conductivity was achieved for the plasticized PEOH4 system. The plasticizer prohibits the formation of ionic crystals by a reduction in columbic interaction, and thereby, the number of free ions increases. Furthermore, it causes rising charge carrier mobility by improving the flexibility of the polymer electrolyte film [64]. The maximum DC ionic conductivity obtained in this study was $1.77\times 10^{-4}$ S cm^−1^ at room temperature for the PEOH4 sample. It is worth noting that the current DC ionic conductivity value is higher than some of the values documented from different works (see Table 3). This means that glycerol plasticizer can improve the ionic conductivity of the PEO-based SPE systems better than those systems used in Table 3.

### 3.2. Transference Number Measurement (TNM)

**Figure 6:**
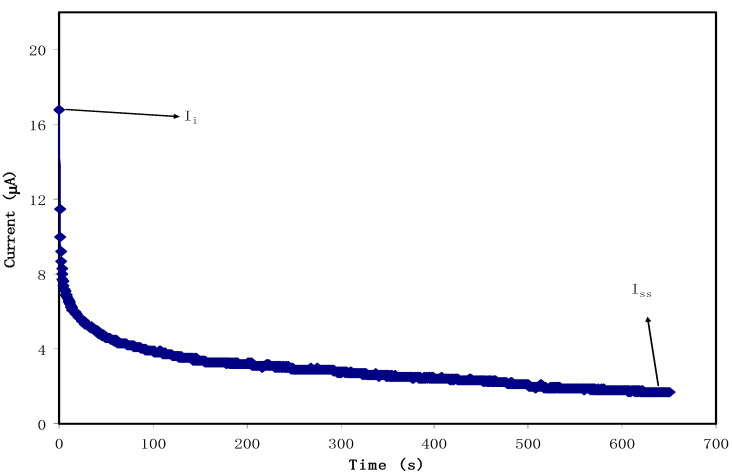
Current–time plot for the highest conducting plasticized (PEOH4) system.

The dominant charge carrier species in polymer electrolytes have been checked by using TNM analysis. The following equations can be used to find the transference number for the ion ($t_{ion}$) and electron ($t_{el}$):

(7) $$t_{ion}=\frac{I_{i}-I_{ss}}{I_{i}}$$

(8) $$t_{el}=1-t_{ion}$$

where $\;I_{i}\;\mathrm{and}\;I_{ss}$ are the initial and steady-state current, respectively. Figure 6 illustrates the polarization curve between current versus time for the maximum conducting plasticized system, which incorporated with 70% PEO:30% NH_4_l:10% glycerol(i.e., PEOH4) at an applied potential of 0.2 V. It demonstrates the maximum value of $I_{i}$ at 17 μA due to both the electrons and ions involvement at the initial stage. The initial current dramatically drops down with time until it gets closer to the steady state at 1.9 μA, after that, gradually decreasing. In this case, the polarization of the cell occurs, and the current flow belongs to electrons instead of ions. This situation indicates that the electrons are the only species that could pass through the stainless steel electrodes while the ions are blocking on it [69]. The values of $t_{ion}$ and $t_{el}$ calculated from Equations (7) and (8) are found to be 0.9 and 0.1, respectively, confirming that the ions are considered as the main charge carrier during the migration process. These results agree with the findings of PEO-NH_4_PF_6_ system [70]. However, in the study of PEO–LiTFSI salt complex with 10 wt. % of different plasticizers (i.e., PG, PC, and EC) by Kim et al., the $t_{ion}$ values were found to be (0.516, 0.262, and 0.381), respectively, which are lower than the present study’s values [37].

### 3.3. Linear Sweep Voltammetry (LSV)

**Figure 7:**
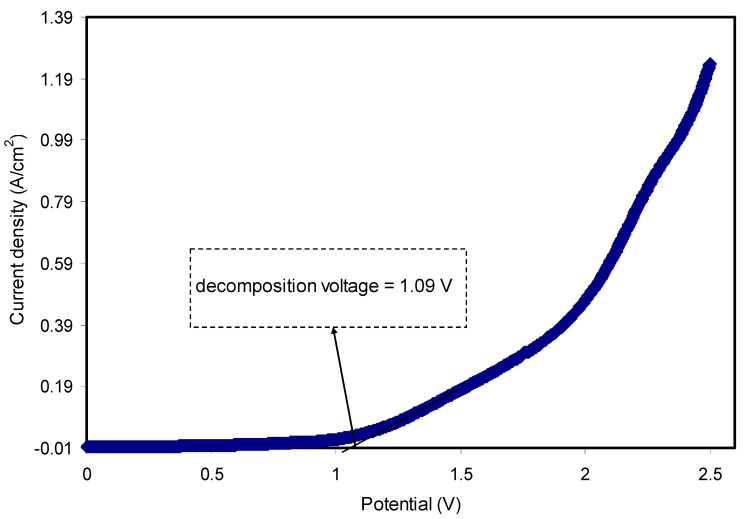
Linear sweep voltammetry for PEOH4 film at scan rate of 10 mV s^−1^ at ambient temperature.

Previous studies confirmed that prior to electrochemical device application, it is crucial to examine the electrochemical stability of the fabricated polymer electrolyte films [71,72,73]. To determine the electrochemical stability and application suitability of polymer electrolytes, LSV was applied for the highest conducting plasticized system, which incorporated with 70% PEO:30% NH_4_l:10% glycerol as shown in Figure 7. The voltage was scanned from 0 to 2.5 V with a scan rate of 10 mV s^−1^ at ambient temperature. It is obvious that no current was detected in the potential range 0–1.09 V, meaning that redox reaction was not taken place in the PEOH4system. Therefore, this system is considered electrochemically at a steady state up to 1.09 V. Beyond 1.09 V, the current increases gradually as a result of breaking down the polymer electrolyte at the inert electrode surface. The applicability of such systems with a standard working voltage of nearly 1 V for proton energy devices has been reported by Pratapet et al. [74]. In comparison, the decomposition voltage for the glycerolized potato starch blended with methylcellulose and doped by NH_4_NO_3_ was 1.88 V at room temperature [56], and for chitosan-PEO-NH_4_NO_3_-EC, it was 1.76 V [75].

### 3.4. Cyclic Voltammetry (CV)

**Figure 8:**
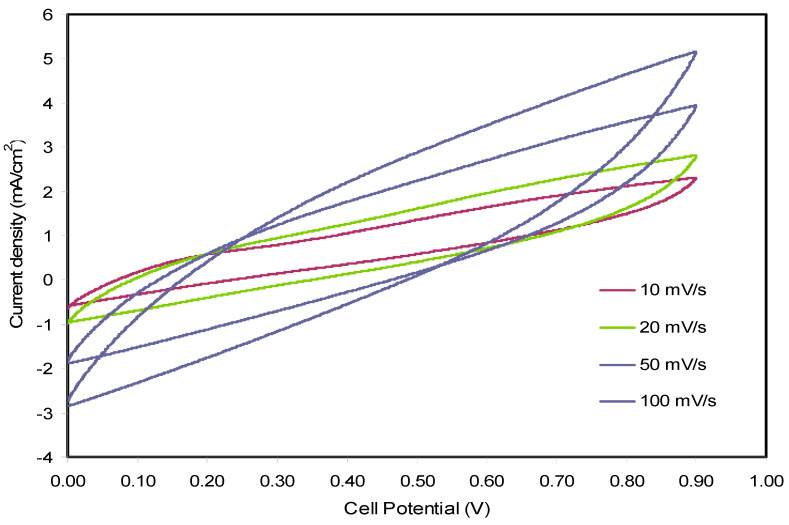
Cyclic voltammetry responses of PEOH4 in the potential range 0 to 0.9 V at different sweep rates from 10 to 100 mV s^−1^.

The electrochemical behaviors such as Faradaic or non-Faradaic reactions and nature of the charge storage of fabricated EDLC at the individual interfaces in the regions of anodic and cathodic are well investigated using CV technique [76,77,78]. In this technique, the current was measured as a function of the potential cell. Figure 8 depicts the CV curves recorded for the PEOH4 electrolyte sample, which incorporated with 70% PEO:30% NH_4_l:10% glycerol at various scan rates. A rectangular-like shape of the curves and no present redox peaks are evidenced for an ideal capacitor. Besides, similar shapes of the curves within scan rates reveal that the potential is independent of capacitor behavior [79,80]. This result also signifies the charge double-layer existence at the activated carbon electrodes surface, hence verifying the non-faradaic process involved in EDLC property [58]. Following the current results, the CV portraits show a rapid response to the applied voltage. All of these are referred to as the insignificant role of electrons and the ion absorption at the electrode–electrolyte interface is, thus, responsible for storing the energy in the EDLC. The specific capacitance (*C_s_*) of the EDLC can be achieved using the profiles of CV at various scan rates due to the following equation:

(9) $$C_{cv}=\int_{V_{i}}^{V_{f}}\frac{I\left(V\right)dV}{2mv\left(V_{f}-V_{i}\right)}$$

where $V_{f}$ and $V_{i\;}$ are the final and initial voltage, respectively. m is the mass of active material,  v  is the scan rates, and ʃ(V)dV is the area of the CV curves. Since the curves in Figure 8 possess different area, the response of the EDLC has relied on the scan rate. Table 4 lists the cell capacitance taken at different scan rates. It seems that the specific capacitance values were decreased with increasing the scan rate resulting from the fact that at a higher scan rate, relatively long charge diffusion length is unable to follow the variation of electric field and high-power density. Moreover, the value of specific capacitance depends on resistance for ion transport, diffusion speed and diffusion length [80,81].

## 4. Conclusions

In the present work, the electrochemical characteristics of synthesized unplasticized and plasticized PEO:NH_4_l electrolyte systems were investigated. When 10 wt. % of glycerol plasticizer was inserted to the highest conducting unplasticized system, the conductivity was increased from $(3.96\times 10^{-5}$ S cm^−1^) to$\;(1.77\times 10^{-4}$ S cm^−1^). The dielectric study data revealed that the dielectric constant was increased at a salt concentration towards the descending frequencies whilst the dielectric modulus was increased towards the ascending frequencies. In addition, the absence of a relaxation peak for PEOH4 system concluded that the increase in conductivity is due to the increase in the concentration of mobile ions. This is attributed to the glycerol plasticizer that causes dissociation of more ammonium iodide to its mobile ions by reason of reducing the columbic interactions between the ions. From the LVS study, the electrochemical stability for the PEOH4 system was evaluated to be up to 1.09 V, indicating its suitability in energy storage device applications. The ion transference number (t_ion_) for this system was determined to be 0.9, proving that ions are the major conducting species. The CV curves for the fabricated EDLC represent non-faradaic electrochemical behavior. Finally, the maximum specific capacitance value for the PEOH4system was found to be 40.46 F g^−1^ at the scan rate of 10 mV s^−1^.

## Figures and Tables

**Table 1:** Composition of both unplasticized and plasticized PEO:NH_4_I systems.

Sample Code	Wt.(g) PEO	Wt. % NH_4_l	Molality (mol/kg)	Wt. % Glycerol
PEOH1	1	10	0.7666	0
PEOH2	1	20	1.7248	0
PEOH3	1	30	2.9568	0
PEOH4	1	30	2.9568	10

**Table 2:** DC Ionic conductivity of un-plasticized and plasticized of PEO:NH_4_I systems at room temperature.

Sample Code	$\mathrm{Ionic Conductivity}\;\sigma_{dc}\;(S\;{\mathrm{cm}}^{-1})$
PEOH1	$1.72\times 10^{-6}$
PEOH2	$2.51\times 10^{-6}$
PEOH3	$3.96\times 10^{-5}$
PEOH4	$1.77\times 10^{-4}$

**Table 3:** Polymer/salt ratio, plasticizer, temperature, and DC conductivity (σ_DC_) for various polymer electrolytes.

Electrolyte Composition	Polymer/Salt Ratio	Plasticizer	T (°C)	σ_DC_ (S cm^−1^)	Ref.
PEO-(NH_4_F)-DMA	F/O = 0.12	DMA	30	$1\times 10^{-4}$	[65]
PEO-[Mg(Cf_3_SO_3_)_2_-EMITF	EO/Mg = ~25	EMITF	~25	$1\times 10^{-4}$	[66]
PEO-LiCf_3_SO_3_-EC	-	EC	23	$1.71\times 10^{-5}$	[67]
(PEO)_8_ LiClO_4_:DBP (99.5:0.5)	(PEO)_8_ LiClO_4_ = 99.5	DBP	29	$5.036\times 10^{-5}$	[68]
PEO:NH_4_I:glycerol	I/O = 2.957	glycerol	29	$1.77\times 10^{-4}$	This work

Where NH_4_F = Ammonium fluoride, DMA = Dimethylacetamide plasticizer, Mg(Cf_3_SO_3_)_2_ = magnesium trifluoromethanesulfonate, EMITF = 1-ethyl-3-methylimidazolium trifluoromethanesulfonate, LiCf_3_SO_3_ = lithium trifluoromethanesulfonate, EC = Ethylene carbonate, LiClO_4_ = Lithium perchlorate, DBP = dibutyl phthalate.

**Table 4:** Specific capacitance values for PEOH4 film at various scan rates.

Scan Rate (mV s^−1^)	Specific Capacitance (F g^−1^)
10	40.46
20	31.43
50	21.03
100	13.46
